# Small B cell lymphocytic lymphoma presenting as obstructive sleep apnea

**DOI:** 10.1186/1477-7819-2-26

**Published:** 2004-07-29

**Authors:** Yung-An Tsou, Yuan-Kai Cheng, Chia-Der Lin, Weng-Cheng Chang, Ming-Hsui Tsai

**Affiliations:** 1 Department of Otolaryngology, China Medical University Hospital, Taichung, Taiwan

## Abstract

**Background:**

Most lymphomas that involve the tonsil are large B cell lymphomas. Large B-cell lymphoma is a high grade malignancy which progresses rapidly. Tonsillar lymphoma usually presents as either a unilaterally enlarged palatine tonsil or as an ulcerative and fungating lesion over the tonsillar area. Small lymphocytic lymphomas (SLL) of the Waldeyer's ring are uncommon.

**Case presentation:**

We report a 41-year-old male who presented with a ten-year history of snoring. Physical examination revealed smooth bilateral symmetrically enlarged tonsils without abnormal surface change or cervical lymphadenopathy. Palatal redundancy and a narrowed oropharyngeal airway were also noted. The respiratory disturbance index (RDI) was 66 per hour, and severe obstruction sleep apnea (OSA) was suspected. No B symptoms, sore throat, odynophagia or dysphagia was found. We performed uvulopalatopharyngoplasty (UPPP) and pathological examination revealed incidental small B-cell lymphocytic lymphoma (SLL).

**Conclusion:**

It is uncommon for lymphoma to initially present as OSA. SLL is an indolent malignancy and is not easy to detect in the early stage. We conclude that SLL may be a contributing factor of OSA in the present case.

## Background

Adenotonsillar enlargement is the main cause of obstructive sleep apnea (OSA) in the pediatric population. However, this prevalent syndrome is more complicated in adults [1]. OSA has also been described in cases of benign lymphoid hyperplasia, plasmacytoma, amyloidosis, pharyngeal tumors and diseases that involve the nasopharyngeal structures. A series of careful examinations of the upper airway should be performed in every adult patient to check for anatomic causes related to upper airway obstruction [2]. We report here a patient with severe obstructive sleep apnea treated by uvulopalatopharyngoplasty (UPPP).

## Case presentation

A 41-year-old man presented with complaints of snoring, excessive daytime sleepiness, and pavor nocturnes for more than 10 years. Systemic diseases were denied. Physical examination revealed bilateral symmetric and enlarged palatine tonsils without abnormal surface change. There were no palpable cervical lymph nodes or B symptoms (fever, body weight loss and cold sweats). White and red blood cell counts, biochemistry and chest radiographs were within normal limits. The results of an overnight polysomnography (PSG) showed mean SaO_2_, 91%, minimal SaO_2_, 62%, and a desaturation index (≥ 4%) of 61.8/h. The arousal index was 64.8/h and the respiratory disturbance index (RDI) was 66.0/h. Believing that the patient was suffering from severe OSA and hyperplastic palatine tonsils, he received UPPP.

**Figure 1:**
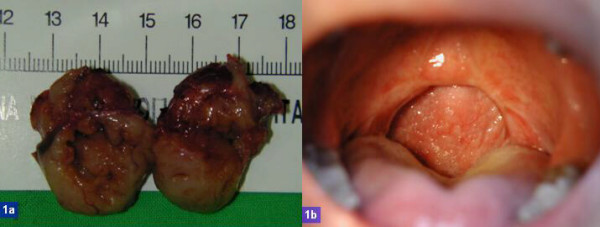
a) surgical specimen of palatine tonsils; b) picture of oropharynx post UPPP 3 months later

**Figure 2:**
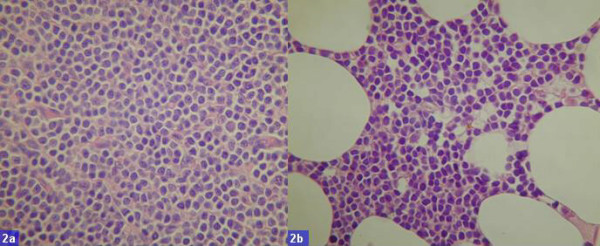
Photomicrograph a) effacement of normal architecture and infiltration of monotonous small lymphoid cells is visible (Hematoxylin and Eosin 100X); b) Bone marrow showing monotonous small lymphoid cells infiltration (Hematoxylin and Eosin 100X).

**Figure 3:**
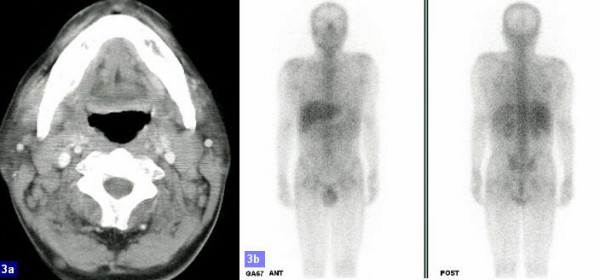
a):Mild lymphadenopathy over bilateral posterior neck area; b:Gallium scan: gallium-avid lymphoma in bilateral submandibular regions and suspected lesions in the mid-abdomen

The postoperative course was uneventful and sleep apnea improved. PSG performed 4 months after surgery demonstrated that the RDI had reduced to 23.9/h. Pathology indicated small B cell lymphocytic lymphoma (Figure 1,2) with bone marrow involvement. During the whole course, the patient was free from B symptoms and no further abnormal lymphadenopathy was detected even after head and neck computed tomography (CT) and thallium scan (figure 3). Chemotherapy was started after evaluation at the oncology clinic. The patient is doing well and is on regular follow-up in the ENT and oncology clinics.

## Discussion

Adenotonsillar enlargement is the leading cause of OSA in the pediatric population [1] though it is not so rare disorder in adults as well. The morbidity of OSA includes hypertension, arrhythmia, heart disease, erythrocytosis, and hyperlipidemia. Malignancy should be considered a potential contributing factor that rarely contributes to OSA and has never been shown to be related to it[2].

Small lymphocytic lymphoma (SLL) is an indolent but relentless malignancy, with a median survival of about 10 years. Because It usually presents as neck lymphadenopathy in the later stages, SLL is not easy to diagnose in the early stage. The effectiveness of chemotherapy for treating SLL is controversial. Most studies have found no benefit in treating patients until they develop symptoms [3]. Lymphoma presenting as OSA is extremely rare, but this case report illustrates that malignancy should be considered a potential contributing factor of OSA; a careful oropharyngeal examination in patients with OSA is necessary. Both tonsillectomy and UPPP can improve the patency of upper airway in OSA patients presenting with abnormally enlarged palatine tonsils. However, pathology of unsuspicious tissues can reveal malignancy with specific staining, and structural abnormalities secondary to a hidden malignancy might present initially as OSA. Therefore, a thorough physical examination should be performed and the pathological results should be closely traced.

Nolan described a case of adenotonsillar enlargement due to chronic lymphatic leukemia which caused severe OSA [4]. His report highlights the need to consider OSA as a cause of constitutional symptoms in adults with lymphoreticular disease, especially when there is involvement of the Waldeyer's ring. Zorick *et al*., [5] reported that upper airway sleep apnea was exacerbated by lymphocytic lymphoma but that chemotherapy led to complete remission of well differentiated lymphocytic lymphoma and subsidence of OSA [5]. Abe *et al*., [6] described a patient with Non-Hodgkin's lymphoma who was successfully treated by tonsillar surgery and chemotherapy. In one published case, complete remission of centrocytic-centroblastic diffuse B cell lymphoma was found after tonsillectomy with UPPP, as in our case [7].

## Conclusions

Tonsillar surgery should be performed even on patients highly suspected of having lymphoma to improve OSA [8-10]. Neck CT is also suggested as a preoperative examination for patients with OSA and neck lymphadenopathy. Whether the prognosis or the outcome of chemotherapy or radiation therapy will be affected by tonsillar surgery is controversial. We conclude that SLL might be a contributing factor of OSA. Therefore careful neck examination should also be performed on patients complaining of snoring or sleep disturbances.

## Competing interest

None declared.

## Authors' contributions

YT, YC, CL, WC and MT made substantial contributions to the intellectual content of the paper, in the interpretation of results and in drafting the manuscript. All authors read and approved the manuscript
